# The complete chloroplast genome sequence of *Barthea barthei* (Melastomataceae), a shrub endemic to southern China

**DOI:** 10.1080/23802359.2017.1403868

**Published:** 2017-11-15

**Authors:** Xuejiao He, Yubing Zhou, Yacheng Cai, Zhendong Chen, Wei Wu, Renchao Zhou, Wei Lun Ng

**Affiliations:** a Fujian Institute of Tropical Crops, Zhangzhou, Fujian, China;; b State Key Laboratory of Biocontrol and Guangdong Provincial Key Laboratory of Plant Resources, School of Life Sciences, Sun Yat-sen University, Guangzhou, Guangdong, China

**Keywords:** *Barthea barthei*, Melastomataceae, endemic species, complete chloroplast genome, automated assembly

## Abstract

The plant genus *Barthea* is monotypic, comprising of only a single species (*Barthea barthei*), and is endemic to southern China. In this study, we report the complete chloroplast genome of *B. barthei*, assembled from whole-genome high-throughput sequencing data, as a resource for future studies on the taxonomy and evolution of *Barthea*. The chloroplast genome was 155,951 bp in length, with a large single-copy (LSC) region of 85,882 bp, a small single-copy (SSC) region of 16,445 bp, separated by two inverted repeat (IR) regions of 26,812 bp each. It was predicted to contain a total of 130 genes, with an overall GC content of 36.99%. Phylogenetic analysis placed *B. barthei* closest to *Opisthocentra* sp. in Melastomataceae.

The plant genus *Barthea* (Melastomataceae), represented by its only species *Barthea barthei*, is endemic to southern China (Chen and Renner 2007). Two varieties of this species are thought to exist, i.e. *B. barthei* var. *barthei* (distributed in Guangdong, Guangxi, Fujian, Hunan, and Taiwan) and *B. barthei* var. *valdealata* (distributed only in Guangxi), differing in several floral and fruit characteristics (Chen and Renner 2007). However, a recent study on populations of both varieties using nuclear microsatellite markers found low genetic differentiation between them, suggesting that the current taxonomic treatment may not hold (Huang et al. 2017). Also, to our knowledge, the taxonomic position of *Barthea* within the family Melastomataceae has not been evaluated phylogenetically. Thus, in this study, we characterized the complete chloroplast genome sequence of *B. barthei* as a resource for future studies on the taxonomy of *Barthea*. We also constructed a phylogeny to confirm its relationship with other genera within the family Melastomataceae.

Sequence data from a whole-genome Illumina paired-end sequencing effort of a *B. barthei* var. *barthei* individual (Huang et al. 2017) sampled from Shenzhen, Guangdong, China (location 22°37′31″N, 114°16′02″E; voucher Q. Fan 14012 deposited in the Sun Yat-sen University Herbarium, SYS), was used for the assembly of this chloroplast genome. Approximately 8 Gb of paired-end (250 bp) sequence data was randomly extracted from the total sequencing output, as input into NOVOPlasty (Dierckxsens et al. 2017) to assemble the chloroplast genome. A partial chloroplast *rbc*L gene sequence of the same species (GenBank accession KX527135) was used as the seed sequence for the seed-and-extend algorithm implemented in NOVOPlasty. Annotation of the chloroplast genome was performed using the Dual Organellar GenoMe Annotator (DOGMA) online tool (Wyman et al. 2004) and Geneious ver. 10.1 (http://www.geneious.com, Kearse et al. 2012), then manually verified and corrected by comparison with sequences of related species on GenBank.

The complete chloroplast genome sequence of *B. barthei* (GenBank accession KY873324) obtained in this study was 155,951 bp in length, with a large single-copy (LSC) region of 85,882 bp, a small single-copy (SSC) region of 16,445 bp, separated by two inverted repeat (IR) regions of 26,812 bp each. It was predicted to contain 130 genes, including 86 protein-coding genes, 36 tRNA genes, and 8 rRNA genes. The overall GC content was 36.99%. The *ndh*D gene had ACG as the start codon, instead of the conventional AUG codon, as reported also in other plant species (Wu et al. 2014).

To investigate the relationship between *Barthea* and other genera within the family Melastomataceae, a phylogenetic tree was constructed. The chloroplast genome of *B. barthei* was aligned with 18 other chloroplast genomes of Melastomataceae (Reginato et al. 2016; Ng et al. 2017) and the *Eucalyptus globulus* chloroplast genome (GenBank accession AY780259) as outgroup, using MAFFT ver. 7.307 (Katoh and Standley 2013). A maximum likelihood tree (Figure 1) was then constructed using RAxML (Stamatakis 2014). *Barthea barthei* appear to be phylogenetically closest to *Opisthocentra* sp.

**Figure 1.**
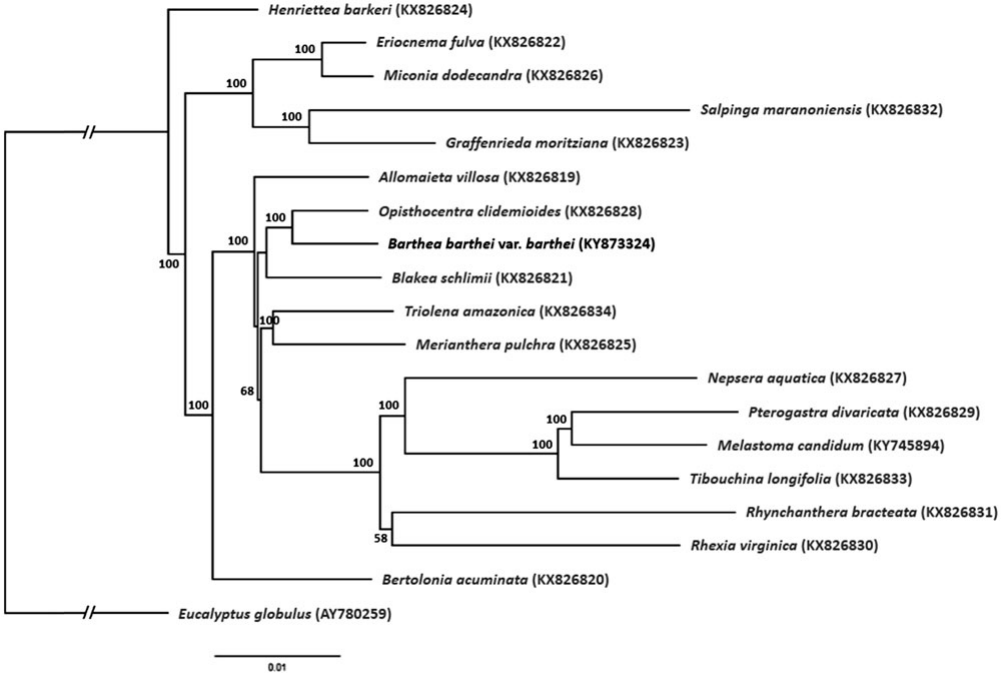
Maximum likelihood tree of Melastomataceae based on complete chloroplast genomes, with *Eucalyptus globulus* as outgroup. Bootstrap support values (based on 1000 replicates) are shown next to the nodes. Scale in substitutions per site.
